# Lingering SARS-CoV-2 in Gastric and Gallbladder Tissues of Patients with Previous COVID-19 Infection Undergoing Bariatric Surgery

**DOI:** 10.1007/s11695-022-06338-9

**Published:** 2022-11-01

**Authors:** Mohamed Hany, Ahmed Zidan, Muhammad Gaballa, Mohamed Ibrahim, Ann Samy Shafiq Agayby, Anwar Ashraf Abouelnasr, Eman Sheta, Bart Torensma

**Affiliations:** 1 Department of Surgery, Medical Research Institute, Alexandria University, 165 Horreya Avenue, Hadara, 21561 Alexandria Egypt; 2 Madina Women’s Hospital, Alexandria, Egypt; 3 Pathology Department, Alexandria University, Alexandria, Egypt; 4 Leiden University Medical Center (LUMC), Leiden, The Netherlands

**Keywords:** Bariatric surgery, Lingering SARS-CoV-2 virus, COVID-19 infection, Long COVID syndrome

## Abstract

**Background:**

Lingering severe acute respiratory syndrome coronavirus 2 (SARS-CoV-2) in gut tissue might be a source of infection during bariatric surgery. This study aimed to confirm the presence of SARS-CoV-2 nucleocapsid in gastric and gallbladder tissues removed during bariatric surgery in individuals previously infected with coronavirus disease 2019 (COVID-19) who had negative polymerase chain reaction results prior to the surgery.

**Methods:**

Gastric and gallbladder specimens from 80 patients who underwent bariatric surgery between November 2021 and May 2022 and had a history of COVID-19 infection with gastrointestinal symptoms were examined for the presence of lingering SARS-CoV-2 nucleocapsid proteins using immunohistochemistry.

**Results:**

Gastric specimens from 26 (32.5%) patients and 4 (100%) cholecystectomy specimens showed positive cytoplasmic staining for the anti-SARS-CoV-2 nucleocapsid protein in surface mucosal epithelial cells. The mean age was 37.8 ± 10.3 years. The average body mass index was 44.2 ± 7.0 kg/m^2^; most of the patients were females (71.3%). The positive staining group was significantly younger than the negative staining group (*p* = 0.007). The full-dose vaccination rate was 58.8%, with a median of 91 days after the last vaccine dose. A positive serological anti-spike IgG response was observed in 99% of the patients. The median time between initial COVID-19 infection and surgery was 274 and 380 days in the positive and negative staining groups, respectively (*p* = 0.371).

**Conclusion:**

Gastric and gallbladder tissues can retain SARS-CoV-2 particles for a long time after COVID-19 infection, handling stomach specimens from patients during an operation must be done with care, as we usually do, but now with the knowledge that in 1/3 of patients they can be present. Performing LSG on post-COVID patients did not seem to increase perioperative morbidity.

**Graphical Abstract:**

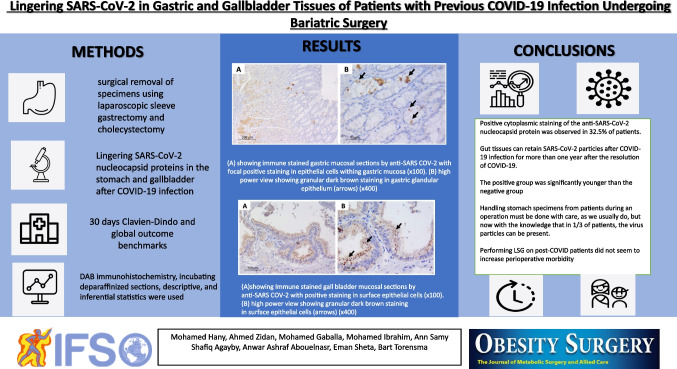

## Introduction

Since the beginning of the coronavirus disease 2019 (COVID-19) pandemic, many studies have been conducted on the general overview, epidemiology, transmission, clinical features, diagnosis, treatment, and clinical outcomes, as well as the prevention and control of COVID-19 [1].

Knowledge regarding the behavior of the virus in human tissue, as well as the presence and effect of COVID-19 in the gastrointestinal tract, is essential. Severe acute respiratory syndrome coronavirus 2 (SARS-CoV-2) in the gut tissue may explain the gastrointestinal symptoms and prolonged shedding of the virus in the feces of patients with COVID-19 [2]. Therefore, it has a direct effect on the perception of the safety of bariatric procedures during the COVID-19 pandemic.

It also serves to debate the safety of bariatric surgeries, where some theories have emerged suggesting that upon using electrocautery and energy devices, the virus might be aerosolized and concentrated in the laparoscopic pneumoperitoneum, which the operating room team might get exposed to [3, 4]. This phenomenon is similar to that of other viruses such as hepatitis B virus [5], human papillomavirus virus [6], and human immunodeficiency virus 1 [7].

Furthermore, the presence of viral material in stool during the active and convalescent phases of COVID-19 suggests the possibility of a fecal–oral route of infection [7].

Since much of the knowledge on the incidence of the viral antigen of SARS-CoV-2 in gastrointestinal tissue is unknown, this study aimed to determine the presence of viral antigens in individuals who were previously infected with COVID-19 and showed a negative polymerase chain reaction (PCR) result.

## Materials and Methods

### Study Setting and Design

This prospective study was conducted between November 2021 and May 2022 at Madina Women’s Hospital, Alexandria, Egypt. It involved the surgical removal of specimens using laparoscopic sleeve gastrectomy (LSG) procedures in patients with previous COVID-19 infection to detect the presence of lingering SARS-CoV-2 nucleocapsid proteins in the stomach and gallbladder.

Secondary outcomes were the 30-day surgical outcomes according to the Clavien-Dindo classification [8], and the Global Outcome Benchmark values for LSG were analyzed [9]

### Patient Selection

Prior to surgery, the electronic medical database was analyzed for records of pre-assessment of patients who had a previous COVID-19 infection, and the patients were contacted to participate. All participants signed an informed consent form for the use of their data for future research. All procedures were performed in accordance with the 1964 Declaration of Helsinki. The study protocol was approved by the Ethics Committee.

### Inclusion Criteria

The inclusion criteria were a history of COVID-19 with one or more gastrointestinal symptoms and a negative PCR test result at the time of bariatric surgery.

### Data Collection

The data retrieved from the medical records included patient demographics, associated medical problems, vaccination status, and the time between the last vaccine dose and surgery. Furthermore, data on COVID-19, including the main symptoms and the duration of illness, were retrieved.

### The Technique of Tissue Examination

**Fig. 1:**
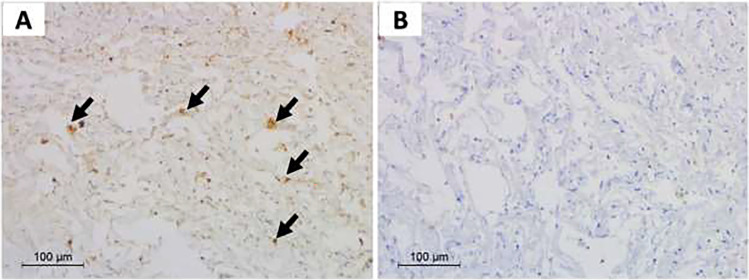
Sections from lung biopsy of a patient with COVID-19. **a** Positive cytoplasmic granular staining after immune staining by anti-SARS-CoV-2 antibody (arrows). **b** Negative staining of the same biopsy when stained by the same technique but with omission of the primary antibody incubation step (× 200)

Gastric and gallbladder specimens were surgically removed, collected immediately after surgery, and fixed overnight in 10% formalin. The following day, the representative sections were processed into paraffin blocks. Hematoxylin and eosin-stained sections were examined to exclude any coincident pathology. Five-micron sections were cut and mounted on positively charged slides. They were stained with anti-SARS-CoV-2 nucleocapsid monoclonal mouse immunoglobulin G (IgG) antibody (clone 1,035,111, Bio-techne, USA, #MAB10474-100) using DAB immunohistochemistry. Immunohistochemistry was performed using an automated Dako autostainer (LINK 48). Antigen retrieval was performed by incubating deparaffinized sections with EDTA solution for 15 min at pH 9. Incubation with primary antibody at a concentration (1:1000) was performed for 30 min. A lung biopsy of a patient infected with COVID-19 (PCR proved) was used as a positive control. Positive staining of pneumocytes lining alveoli was observed. Negative control was obtained by omitting the primary antibody incubation step during the same lung biopsy (Fig. 1).

### Quantification of SARS‑CoV‑2 Antibody Response

According to the manufacturer’s instructions, Sera were separated and tested on the commercially available Elecsys® Anti-SARS-CoV-2 assay (Roche Diagnostics International Ltd, Rotkreuz, Switzerland) [10]. The Elecsys Anti-SARS-CoV-2 S is a quantitative serologically that detects high-affinity antibodies to the SARS-CoV-2 S protein RBD and has a low risk of detecting weakly cross-reactive and non-specific antibodies. Quantitative antibodies against SARS‑CoV‑2 RBD with a strong neutralizing capacity were strong positive (> 10 U/mL).

#### LSG

The LSG procedure team performed the LSG procedures throughout the study. Dissection began 6 cm from the pylorus (antrum preserving) until the gastroesophageal junction, followed by gastric transaction over a 40F bougie through sequential stapler firings.

### Statistical Analysis

Descriptive and inferential statistics were used. All data were first tested for normality using the Kolmogorov–Smirnov test, *Q*–*Q* plot, and Levene’s test. Categorical variables were expressed as *n* (%). Continuous normally distributed variables were represented as mean and standard deviation, and non-normally distributed data were represented as median and interquartile range (IQR) for skewed distributions. To compare categorical variables among different groups, we used Pearson’s chi-square test or Fisher’s exact test when appropriate. Normally distributed continuous data were tested with independent samples, and the student’s *t*-test was used to compare continuous variables between independent samples. For skewed data, the Mann–Whitney *U* test was used. Statistical significance was set at *p* ≤ 0.05. Statistical analyses were performed using R software version 4.1.3.

### Sample size calculation

Since the incidence of SARS-CoV-2 in tissues is still unknown, we conducted several power scenarios. With a power of 80% and an alpha of 0.05 as a basis, in scenario 1, for a delta of 0.4 (0.7 vs. 0.3 indicating COVID-19 is absent vs. present), 11 samples were necessary; in scenario 2, for the delta of 0.2 (0.6 vs. 0.4), 48 samples were necessary, while in scenario 3, for the delta of 0.16 (0.58 vs. 0.42), 75 samples were necessary. We chose the most conservative power scenario, scenario 3, and collected 80 samples from 80 patients (1 sample per patient). R software version 4.1.3 and its “pwr” package was used.

## Results

This study included 80 patients who underwent LSG, of whom four (5%) underwent concomitant cholecystectomy for chronic calcular cholecystitis.

The mean age was 37.8 ± 10.3 years, and most of them were females (71.3%). The average body mass index of the patients was 44.2 ± 7.0 kg/m^2^. The most commonly associated medical conditions were diabetes, dyslipidemia, hypertension, apnea, and asthma. Multiple associated medical conditions occurred in 55.5% of the patients (Table 1).

**Table 1:** Patient characteristics, comorbidities, and vaccination status

	Positive SARS-CoV-2 Tissue staining (*N* = 26)		Negative SARS-CoV-2 Tissue staining (*N* = 54)		*P* value
	*N*	%	*N*	%	
Sex (female)	18	69.2%	39	72.2%	0.797
Age in years (M ± SD)	33.6	± 8.4	39.8	± 10.6	0.007
Weight in kg (M ± SD)	124.4	± 20.1	119.8	± 22.0	0.358
BMI in kg/m^2^ (M ± SD)	44.8	± 5.5	43.9	± 7.6	0.548
Associated medical problems					
Multiple associated medical problems	12	46.2%	35	64.8%	0.178
Any associated medical problems	12	46.2%	35	64.8%	0.147
Diabetes	2	7.7%	8	14.8%	0.486
Dyslipidemia	5	19.2%	12	22.2%	1
Hypertension	6	23.1%	15	27.8%	0.789
Apnea	4	15.4%	11	20.4%	0.763
Asthma	2	7.7%	3	5.6%	0.658
Renal transplant	1	3.8%	0	0.0%	0.325
Hypothyroidism	1	3.8%	5	9.3%	0.658
Cardiovascular	1	3.8%	2	3.7%	1
Gout	0	0.0%	1	1.9%	1
Rheumatoid	0	0.0%	1	1.9%	1
GERD	0	0.0%	1	1.9%	1
Vaccination status & doses					0.626
None	9	34.6%	17	31.5%	
Partial	1	3.8%	6	11.1%	
Complete	16	61.5%	31	57.4%	
Time from last vaccine dose to surgery (days), Median (IQR)^a^	61 (92)		91 (122)		0.216

^a^Comparison between the 17 vaccinated tissue-positive cases and the 37 vaccinated tissue-negative cases

SARS-CoV-2, severe acute respiratory syndrome coronavirus 2; M ± SD, mean ± standard deviation; IQR, interquartile range; BMI, body mass index; GERD, gastroesophageal reflux disease

### Tissue Staining

In total, 26 patients (32.5%) showed positive cytoplasmic staining for anti-SARS-CoV-2 nucleocapsid protein (positive group), and 54 (67.5%) showed negative cytoplasmic staining (negative group).

**Fig. 2:**
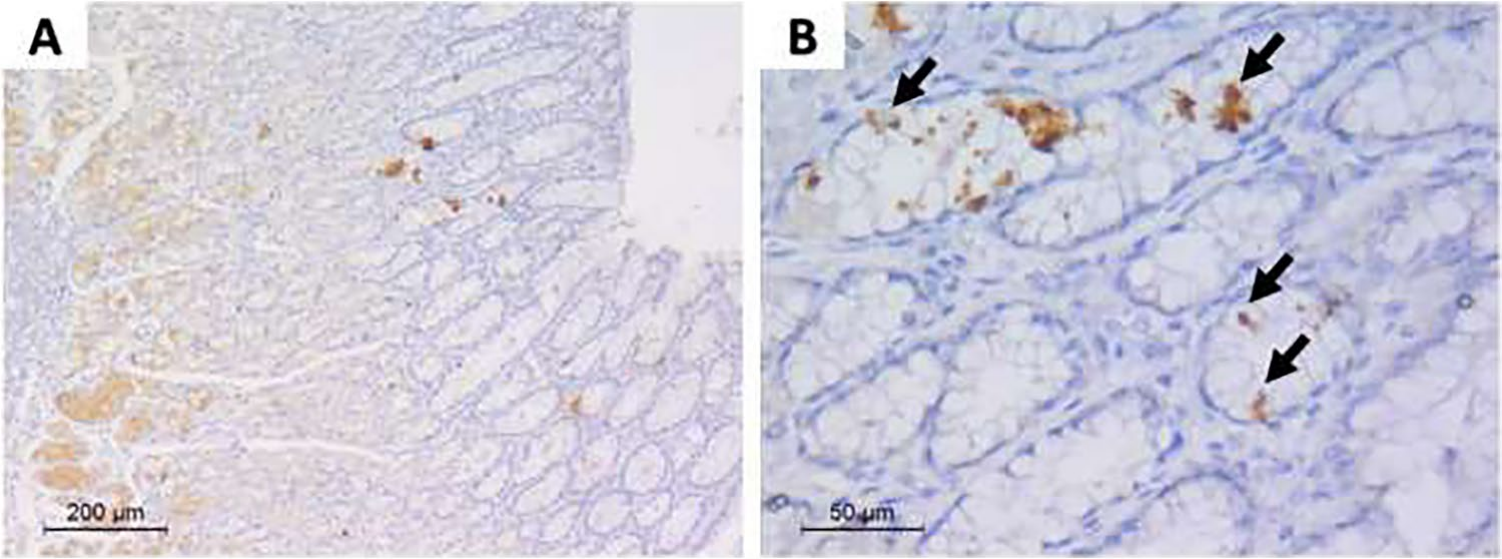
Sections from gastric biopsy. **a** Immune-stained gastric mucosal sections by anti-SARS-CoV-2 antibody, with focal positive staining in epithelial cells within the gastric mucosa (× 100). **b** High-power view showing granular dark brown staining in the gastric glandular epithelium (arrows) (× 400)

Pathology results showed that the positive group had dark brownish granules in the cytoplasm of the epithelial lining. It was observed in the surface epithelium and the lining of superficial glands (Fig. 2).

### Symptoms During Infection

Both groups showed fever, headache, and malaise during the COVID-19 infection, but there were no significant differences (34.6% vs. 51.9%, 3.8% vs. 7.4%, and 38.5% vs. 46.3%, respectively; *p* = 0.161, 1.00, and 0.632, respectively) (Table 2).

**Table 2:** Type and duration of symptoms experienced during COVID-19 infection

	Positive SARS-CoV-2 tissue staining (*N* = 26)		Negative SARS-CoV-2 Tissue staining (*N* = 54)		*P* value
	***N***	**%**	***N***	%	
Symptoms during infection					
Fever	9	34.6%	28	51.9%	0.161
Headache	1	3.8%	4	7.4%	1
Malaise	10	38.5%	25	46.3%	0.632
Respiratory symptoms	15	57.7%	33	61.1%	
Cough	9	34.6%	17	31.5%	0.803
Dyspnea	8	30.8%	21	38.9%	0.621
Hypoxia	2	7.7%	5	9.3%	1
Sore throat	1	3.8%	1	1.9%	0.547
Gastrointestinal symptoms					
1 or more symptoms	26	100%	54	100%	-
Ageusia and anosmia	18	69.2%	33	61.1%	0.621
Abdominal pain	11	42.3%	17	31.5%	0.453
Diarrhea	9	34.6%	21	38.9%	0.808
Anorexia	0	0.0%	1	1.9%	1
Vomiting	9	34.6%	20	37.0%	1
Thrombosis	1	3.8%	0	0.0%	0.325
Duration of symptoms (days), Median (IQR)	14 (1.5)		14 (4)		0.793
Time from COVID-19 infection to surgery (days), Median (IQR)	274 (213)		380 (288)		0.371

COVID-19, coronavirus disease 2019; SARS-CoV-2, severe acute respiratory syndrome coronavirus 2; IQR, interquartile range

### Respiratory Symptoms

Both groups showed coughing, dyspnea, hypoxia, and sore throat, but there were no significant differences (34.6% vs. 31.5%, 30.8% vs. 38.9%, 7.7% vs. 9.3%, and 3.8% vs. 1.9%, respectively; *p* = 0.803, 0.621, 1, 0.547). (Table 2).

### Gastrointestinal Symptoms

During the COVID-19 infection, all patients (100%) with COVID-19 in both groups had one or more gastrointestinal symptoms. Ageusia and anosmia were present in 69.2% and 61.1% of the positive and negative groups, respectively (*p* = 0.621). Diarrhea occurred in 34.6% and 38.9% (*p* = 0.808) of the respective groups, anorexia in only 1 patient in the negative group (*p* = 1.00), vomiting in 34.6% and 37.0% (*p* = 1.00) of the respective groups, and thrombosis in only 1 patient in the positive group (*p* = 0.325). No differences were found in the duration of symptoms (median [IQR]: 14 [1.5] vs. 14 [4] days; *p* = 0.793) (Table 2).

### Vaccination

In total, 47 patients (58.75%) were fully vaccinated and 7 (8.75%) received a single-dose vaccination. Pfizer, Sinopharm, and Oxford/AstraZeneca COVID-19 vaccines were used. Twenty-six patients (32.5%) were not vaccinated.

Sixteen patients in the positive group (61.5%) and 31 patients (57.4%) in the negative group were fully vaccinated (> 2 doses of Pfizer miRNA vaccines). None of the vaccination subgroups were significantly different from the positive and negative groups (*p* = 0.626), and no significant differences were identified between the vaccination types in both groups (*p* = 0.978).

The median (IQR) time since the last vaccine dose was 61 (92) days and 91 (122) days in the positive and negative groups, respectively (*p* = 0.216). The time between SARS-CoV-2 infection and surgery was 274 (213) and 380 (288) days, respectively (*p* = 0.371). (Table 2).

### Positive Tissue Staining

The group with positive tissue staining was significantly younger, with a mean of 6 years (95% confidence interval: 1.8 to 10.5) compared with the negative staining group (*p* = 0.007). Furthermore, no significant differences were found between the two groups in terms of anthropometrics and associated medical problems.

### Anti-spike IgG

Strong positive serological anti-spike IgG responses (> 10 U/mL) were observed in 99% of all patients (positive and negative groups); only one non-vaccinated patient in the positive group had a negative serological response (< 1 U/mL).

### Cholecystectomy

During the LSG operation, a concomitant cholecystectomy was performed in 4 (5%) out of a total of 80 (R2A3) patients, all from the positive group. All four patients showed positive expression of SARS-CoV-2 nucleocapsid proteins.

None of the patients (0%) in the negative group underwent cholecystectomy.

**Fig. 3:**
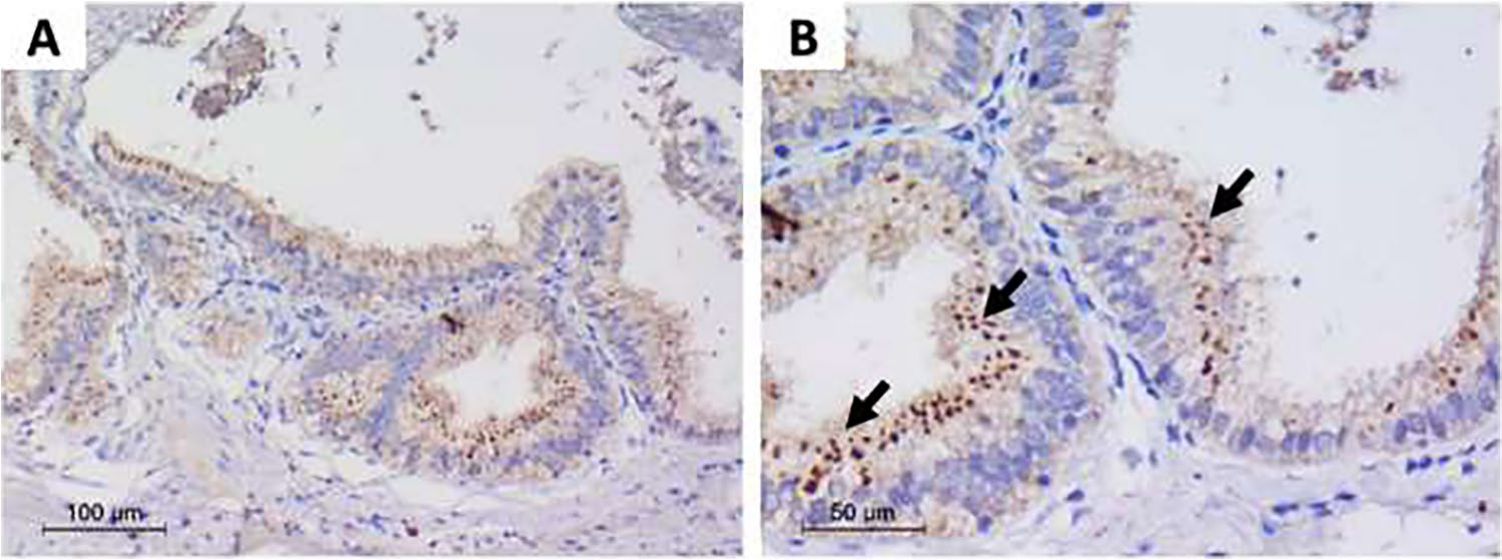
Sections from gallbladder biopsy. **a** Immune-stained gallbladder mucosal sections by anti-SARS-CoV-2 antibody, with positive staining in surface epithelial cells (× 100). **b** High-power view showing granular dark brown staining in surface epithelial cells (arrows) (× 400)

Pathology results showed gallbladder expression mainly in the surface mucosal epithelial cells. It was diffuse and strong compared to gastric biopsies (Fig. 3).

### Complications After LSG and Global Outcome Benchmarks

One patient (1.25%) scored Clavien-Dindo (CD) grade 1 regarding excessive vomiting after the LSG. This patient was treated with intravenous medication and fluids in the outpatient clinic and discarded the same day. Furthermore, no complications were registered. 0% of the patients had a CD ≥ grade 2, and there was no correlation between the COVID-19 virus in tissue and the postoperative complication.

The Global Outcome Benchmark (GOB) values in bariatric surgery for LSG showed 72.8% of benchmark cases.

## Discussion

This prospective study tested surgically removed specimens using LSG procedures in patients with previous COVID-19 infection to detect the presence of lingering SARS-CoV-2 nucleocapsid proteins in tissues.

The incidence was 32.5% for positive cytoplasmic staining of the anti-SARS-CoV-2 nucleocapsid protein and 67.5% for negative cytoplasmic staining.

To the best of our knowledge, this is the first study to examine specimens from the stomach or gallbladder in the presence of SARS-CoV-2 after a COVID-19 infection. This new knowledge could help us better understand the behavior of SARS-CoV-2, thereby impacting the healthcare practice and patient treatment.

SARS-CoV-2 particles have been detected in tissues from different organs in the body for months after the resolution of the initial COVID-19 infection. They have also been linked to the long COVID syndrome [4, 11–13].

Viral RNA and proteins can be found in gut tissues, such as the colon, intestine, duodenum, appendix, bile, and peritoneal fluid several months after infection [4, 11, 13].

Viral RNA has also been found in other organs outside the gut, including the central nervous system, heart, eyes, brain (up to 230 days after infection), and breasts [14]. Therefore, this study confirmed that even after a long recovery period, SARS-CoV-2 particles were found in 99% of the patients with an anti-spike IgG response detected in the blood.

### Global Outcome Benchmark and Complications

The Global Outcome Benchmarks (GOB) values in bariatric surgery for LSG showed 72.8% of benchmark cases. The GOB presented that the proportion of benchmark cases within the case mix of participating centers varied from 4 to 69% [9]. Our study had a 0% Clavien-Dindo grade ≥ 2 (and one, (1.25%) CD grade 1).

The GOB values stated that any complications within 30 days should be ≤ 11%, for complication grade ≥ IIIa must be < 5%, and signature complications like staple line leak, dysphagia/stenosis of the gastric tube, postoperative bleeding, small bowel obstruction, and wound infection should be below 0.3–2.2%. Performing LSG on post-COVID patients did not seem to increase the perioperative morbidity. Nevertheless, this study cohort had only 80 inclusions, and therefore possibly underpowering and underestimating the GOB would be possible.

### Positive Tissue Staining

In this study, we found a significant difference in age between the positive and negative groups, with the positive staining group being significantly younger. Data published in the literature do not show a relationship between age and the detection of lingering SARS-CoV-2 [4, 11–13, 15]. Other factors have been identified as predictors for long COVID-19, including diabetes mellitus, but not young age [16].

Some studies have shown durable antibodies against SARS-CoV-2 for 1 year after COVID-19 [17]. The strong positive serological anti-spike IgG response in our study could be related to the initial COVID-19 infection, vaccination, or recent subclinical reinfection. This study showed a strongly positive serological anti-spike IgG response at the surgery. The median time between COVID-19 and surgery was 274 days and 380 days in the positive and negative groups, respectively, confirming the effect of antibodies on normal blood outcomes after a relatively long period. The correlation between the effect of antibodies and detection of lingering SARS-CoV-2 is difficult to determine and remains unclear. Ninety-nine percent of all patients had antibodies, and only 32.5% had positive cytoplasmic staining for anti-SARS-CoV-2. Furthermore, no significant differences in the vaccination status were observed between the positive and negative groups.

Some studies on other viruses have tested the effects of tissue reservoirs as a source of viruses for a long time [18, 19]. However, this remains to be investigated in the context of COVID-19. A systematic review by Cheruiyot et al. reported the detection of SARS-CoV-2 in abdominal tissues, fluids, and surgical smoke [4]. SARS-CoV-2 RNA was identified in 11 of 29 patients (37.9%). The largest included study was a case series of five surgical patients (bowel resection, appendectomy, rectosigmoid resection, and drainage of hemoperitoneum) [20]. Additionally, these percentages are consistent with the results of the present study (32.5%). Even with a much larger study power, 3 out of 10 patients developed positive cytoplasmic staining for anti-SARS-CoV-2. The etiological pathways of COVID-19 and positive cytoplasmic staining of anti-SARS-CoV-2 should be further investigated for possible tissue reservoir’ effects on SARS-CoV-2 in the tissues, as well as for possible negative effects on the body, therapy, and revival of the virus.

### Validation of Identifying SARS-CoV-2 in Tissues

In this study, immunohistochemistry was used to detect SARS-CoV-2 in stomach and gallbladder tissues using anti-SARS-CoV-2 nucleocapsid monoclonal antibodies. Most studies have identified the presence of SARS-CoV-2 by testing for viral RNA using reverse transcriptase (RT)—PCR. This testing technique has been shown to yield false-negative results [4, 11, 13, 21, 22]. Some studies have reported immunostaining with polyclonal and monoclonal antibodies against SARS-CoV-2 nucleocapsid proteins to detect SARS-CoV-2 nucleocapsid proteins in the cytoplasm of the cells of the gut [15], adipose tissue, along with RT-PCR [23]. Immunostaining may be more accurate because it detects viral particles in the cytoplasm of cells. Moreover, PCR has the theoretical risk of sample contamination with false-positive results [4].

Basolo et al. detected the presence of the virus in all samples that tested positive using RT-PCR [23]. Zollner et al. detected positive immunostaining of nucleocapsid proteins only in the gut mucosa of 24 patients, while RT-PCR results were positive for the gut mucosa samples of 32 patients [15]. Other authors have reported negative RNA in some patient tissue samples that tested positive by immunohistochemistry staining [24]. The staining patterns of gastric and gallbladder tissues in the present study were similar to those reported by Gray‐Rodriguez et al. during active SARS-CoV-2 infection, with focal involvement of the surface epithelium and superficial glands lining cells in the stomach and diffuse and strong involvement in the gallbladder epithelium, which may be correlated to the expression of ACE2 receptors on cells [25]. Notably, virus cultivation from the same biopsies failed in all studies, indicating the absence of infectious viruses [15, 23].

Upon reviewing the literature, we found that no previous immunohistochemical staining of gut tissues for frequent infectious diseases, such as the influenza virus, enteric adenovirus, or cytomegalovirus, has been carried out in gut specimens. Only faecal shedding and PCR testing have been researched [26–33]. To our knowledge, our study showed the presence of a virus (SARS-CoV-2) in gastric specimens and in the gall bladder post-bariatric surgery, for the first time.

### Implications to the Future

The currently available studies could not prove the presence of living viruses in tissue samples using viral culture to assess the infectivity of gut tissue samples containing viral RNA after acute COVID-19 [15, 23]. Even if there is any correlation between the infectivity of these tissue specimens over time and the diagnoses and incidence of “long-COVID” disease, it has not yet been proven.

Research on COVID-19 was new, and a lot has been written about the virus and the disease in the past years. However, not everything has been highlighted yet. On August 1, 2020, a modified Delphi Consensus protocol was created by a group of experts in the bariatric field on what needs to be done after resuming bariatric and metabolic surgery during the COVID-19 pandemic. All factors were taken into consideration, even with regard to instances where evidence was not available, such as, for example, surgical smoke, and a logical consensus was created [34].

A systematic review published after this Delphi Consensus protocol was established revealed conflicting results relating to the presence or absence of SARS-COV-2 RNA in abdominal tissues and fluids. The essential concerns were that the technique of testing for COVID-19 generated false negative results 40% of the time and that the studies presented did not test any form of viral culture or conduct cytopathic studies [4]. We can conclude now, with new research, that surgical smoke does not pose any risk [35–37]. The goals of all the research and consensus manuscripts were the protection of patients and operation theatre staff in and around these patients.

This process was followed by our study which provided proof, for the first time, that COVID-19 virus remnants are still present in the tissues of 1/3 of patients after one year or more. However, we cannot confirm what this implies for viral loading, spread, or contamination. Until new research concludes otherwise, attention and focus are still necessary.

To determine whether elective surgery for patients with obesity opt for a bariatric procedure (R2A2) should be avoided for a certain period after COVID-19 infection, and to understand the possible risks for patients, several studies have demonstrated when to resume elective surgery after recovery from COVID-19. A study by COVIDSurg [40] found that a delay of ≥ 7 weeks after COVID-19 could reduce the mortality rate in cases where the symptoms had resolved, or the patient had been asymptomatic. This COVIDSurg study focuses on all types of surgery and is not specific to bariatric surgery. Furthermore, criteria such as age (> 70), cancer, emergency surgery, and a cardiac risk index of > 3 were associated with an increased chance of mortality, and patients meeting the aforementioned criteria are, therefore, not the main target population for bariatric surgery. Additionally, the group with no COVID-19 signs had a postoperative mortality rate of 1.4%. When we compared this with bariatric surgery post-operative mortality rates studied in 41,241 primary bariatric procedures, the 30-day mortality rate after discharge was 0.08% and in-hospital death rate after surgery was 0.07% [41]. However, the COVIDSurg study provides a good account of the effects of surgery after COVID-19 and of the timeline involved. A study specific to bariatric surgery obtained a safety outcome indicating that minor and moderate COVID-19, especially the forms not complicated with invasive mechanical ventilation, should not preclude indications for bariatric surgery. It was noted that this held true where the mean interval from SARS-CoV-2 infection to bariatric surgery was 11.3 weeks (3–34) [42]. Another study found that bariatric surgery was safely performed in patients who had made a full recovery from COVID-19 without increased complications due to cardiovascular or pulmonary venous thromboembolisms increasing the mortality rate (which was 0%). The average time from a COVID-19 diagnosis to surgery was 82.5 days for the entire cohort (range 12–290), 54 days for asymptomatic patients, and 102.5 days for mildly symptomatic patients [43]. Hence, given COVIDSurg’s advice of ≥ 7 weeks (100% fulfilled this criterion in our study) and the bariatric surgery study’s suggestion of an average of 11–13 weeks for asymptomatic patients, a minimum delay of not less than seven weeks but more towards 11–13 weeks is advisable. Furthermore, in our experience, prior COVID-19 does not induce additional specific complications following bariatric surgery.

### Limitations

We could not assess the infectivity of these viral particles in tissues. Assessment of infectivity by detection of live SARS-CoV-2 in specimens using cell cultures would be of great value in the future. Furthermore, all included patients had negative PCR results at the time of surgery; therefore, the possibility of reinfection at the time of surgery cannot be ruled out completely. Finally, since the study included 80 patients using the most conservative power, it was deemed sufficient. Nevertheless, to obtain more reliable answers and possible corrections for bias and confounding factors, more tissues should be included.

## Conclusion

Positive cytoplasmic staining of the anti-SARS-CoV-2 nucleocapsid protein was observed in 32.5% of patients. Additionally, gastric and gallbladder tissues can retain SARS-CoV-2 particles for more than one year after the resolution of COVID-19. Furthermore, performing LSG on post-COVID patients did not seem to increase perioperative morbidity. Handling stomach specimens from patients during an operation must be done with care, as we usually do, but now with the knowledge that in 1/3 of patients, virus particles can be present. In COVID-19-positive cases, prolonged multi-organ involvement with virus particles was found. These may be responsible for the long COVID syndrome or late postoperative complications.
